# Designer umbilical cord-stem cells induce alveolar wall regeneration in pulmonary disease models

**DOI:** 10.3389/fimmu.2024.1384718

**Published:** 2024-04-30

**Authors:** Mayumi Iwatake, Tokiko Nagamura-Inoue, Ryoichiro Doi, Yukinori Tanoue, Mitsutoshi Ishii, Hiroshi Yukawa, Keitaro Matsumoto, Koichi Tomoshige, Takeshi Nagayasu, Tomoshi Tsuchiya

**Affiliations:** ^1^ Institute of Nano-Life-Systems, Institutes of Innovation for Future Society, Nagoya University, Nagoya, Japan; ^2^ Division of Surgical Oncology, Department of Surgery, Nagasaki University Graduate School of Biomedical Sciences, Nagasaki, Japan; ^3^ Department of Cell Processing and Transfusion, The Institute of Medical Science, The University of Tokyo, Tokyo, Japan; ^4^ Department of Thoracic Surgery, Faculty of Medicine, Academic Assembly, University of Toyama, Toyama, Japan

**Keywords:** cell adhesion, cellular therapy, COPD, macrophage, osteoporosis

## Abstract

**Background:**

Researchers are focusing on cellular therapy for chronic obstructive pulmonary disease (COPD) using mesenchymal stem cells (MSCs), with human bone marrow-derived MSCs (hBM-MSCs) leading the way. However, BM-MSCs may not be as optimal as therapeutic cells owing to their low growth potential, invasive harvesting, and high expression of aging-related genes with poor differentiation potential. Consequently, umbilical cord-derived MSCs (hUC-MSCs), which have many excellent features as allogeneic heterologous stem cells, have received considerable attention. Allogeneic and heterologous hUC-MSCs appear to be promising owing to their excellent therapeutic properties. However, MSCs cannot remain in the lungs for long periods after intravenous infusion.

**Objective:**

To develop designer hUC-MSCs (dUC-MSCs), which are novel therapeutic cells with modified cell-adhesion properties, to aid COPD treatment.

**Methods:**

dUC-MSCs were cultured on type-I collagen gels and laminin 411, which are extracellular matrices. Mouse models of elastase-induced COPD were treated with hUC-MSCs. Biochemical analysis of the lungs of treated and control animals was performed.

**Results:**

Increased efficiency of vascular induction was found with dUC-MSCs transplanted into COPD mouse models compared with that observed with transplanted hUC-MSCs cultured on plates. The transplanted dUC-MSCs inhibited apoptosis by downregulating pro-inflammatory cytokine production, enhancing adhesion of the extracellular matrix to alveolar tissue via integrin β1, promoting the polarity of M2 macrophages, and contributing to the repair of collapsed alveolar walls by forming smooth muscle fibers. dUC-MSCs inhibited osteoclastogenesis in COPD-induced osteoporosis. hUC-MSCs are a promising cell source and have many advantages over BM-MSCs and adipose tissue-derived MSCs.

**Conclusion:**

We developed novel designer cells that may be involved in anti-inflammatory, homeostatic, injury repair, and disease resistance processes. dUC-MSCs repair and regenerate the alveolar wall by enhancing adhesion to the damaged site. Therefore, they can contribute to the treatment of COPD and systemic diseases such as osteoporosis.

## 1 Introduction

Researchers are developing cell-based therapies for lung diseases. The life-saving effects of mesenchymal stem cell (MSC) therapy for patients with COVID-19-ARDS have been well documented, and reports on the use of cell-based therapies in preclinical experimental models of chronic obstructive pulmonary disease (COPD) have been published (1, 2). Clinical trials exploring the use of allogeneic bone marrow-derived MSCs (BM-MSCs) for the treatment of COPD are underway in the U.S. and India, with documented evidence of the anti-inflammatory effects of BM-MSCs (3, 4). However, BM-MSCs are not preferred as therapeutic cells because of their low proliferative potential and susceptibility to invasive infections in recipients of transplanted BM-MSCs. BM-MSCs express human leukocyte antigen (HLA) class II molecules that induce immune reactions and inflammation (5).

Umbilical cord-derived MSCs (UC-MSCs) are suitable for allogeneic transplantation because they do not express HLA-II and show high immune tolerance (6, 7). Umbilical cord tissues can be collected noninvasively and stored in banks to ensure a stable supply of UC-MSCs. UC-MSCs exhibit greater immunosuppression than BM-MSCs due to the secretion of soluble factors (prostaglandin E2 and galectin-1) and their high adherence ability (8).

Administration of human UC-MSCs (hUC-MSCs) for COPD therapy has been clinically proven to be beneficial (9–11). Paracrine action might underlie the improvement observed following MSC administration, but it does not promote lung reconstruction (repair of alveolar epithelial cells) (12). Therefore, cells that effectively promote tissue regeneration must be developed. By standardizing the culture conditions for hUC-MSCs to optimize their diverse functions, it is possible to create therapeutic cells for COPD and ARDS treatment that stimulate vascular induction and possess anti-inflammatory properties.

Modulation of hUC-MSCs induces the formation of adhesion structures that connect cells to the extracellular matrix (ECM), whereas cytoskeletal actin modulation promotes osteoblast differentiation and neovascularization (13, 14). Interstitial matrix proteins, including collagen and fibrin/fibronectin, serve as key receptors that interact with endothelial cell (EC) surface integrins to activate ECs (15–18). Therefore, the objective of the study was to develop designer hUC-MSCs (dUC-MSCs) by modifying their ECM.

COPD coexists with various systemic diseases including osteoporosis, hyperlipidemia, hypertension, and ischemic heart disease. Here, we focused on osteoporosis, one of the major complications of COPD, and analyzed the contribution of dUC-MSC to osteoclastogenesis in a COPD mouse model. Our findings provide new insights into the immunomodulatory functions of dUC-MSCs used to repair lung injury in COPD mouse models that could be used for the treatment of ARDS.

## 2 Materials and methods

### 2.1 Cell culture

hUC-MSCs were provided by Dr. Tokiko Nagamura (Tokyo University, Tokyo, Japan). A two-dimensional collagen gel (Type I-A, Nitta Gelatin Inc. Nitta Gelatin, Tokyo, Japan) culture system was prepared following the manufacturer’s protocol. The collagen gel solution containing hUC-MSCs was poured into a plastic Petri dish, followed by incubation at 37°C to allow the gel to polymerize. Subsequently, hUC-MSCs were seeded onto the polymerized gels.

In this study, umbilical cord MSCs were seeded after coating plastic culture dishes with fibronectin, collagen gel, collagen coat, Laminin 211, Laminin 411, and Laminin 511 ECM. The cells were characterized by seeding umbilical cord MSCs in different culture environments created using these techniques. Normal culture without ECM was described as non-coated.

In addition, plates coated with a mixture of collagen and Laminin 411 were described as collagen coat-L411, in which 1 mL of diluted collagen concentration was mixed with 9 μl of Laminin 411, added to the wells and allowed to stand at room temperature for 1 hour. The collagen was used as collagen coat-L411. Gel-L411 plates were also prepared and used by adding 9 μl of Laminin 411 to 2 mL of dilute collagen solution, mixing, adding to wells, and heating at 37°C for 30 minutes to gelatinize.

The cells from the Gel and Gel-L411 culture environment were lysed from the gel using collagenase and used for gene expression analysis and flow cytometry. Cells from other culture methods were exfoliated using trypsin.

### 2.2 Animal model of COPD

C57BL/6 (B6) mice were purchased from CLEA Japan Inc. (Tokyo, Japan) and maintained under conventional conditions at the Nagasaki University Animal Center. Induction of emphysema was performed according to a previous protocol established as previously reported (19, 20). Briefly, the animals were treated with 3% isoflurane under oxygen anesthesia and challenged with intranasal instillation of 30 μg porcine pancreatic elastase (catalog number 058-05361; FUJIFILM Wako Pure Chemical Corporation, Osaka, Japan) in 50 µl of 0.9% saline solution. The animals were administered the dose only once on Day 0. Control animals were administered 50 µl of 0.9% saline solution (vehicle). Lung samples collected from treated and control animals were analyzed (see online **Data Supplement** for details).

### 2.3 hUC-MSC administration

The mice were anesthetized (isoflurane: 3% induction and 1% maintenance), and saline solution or cultured hUC-MSCs (3.0 × 10^6^ cells, total volume 100 μl) were slowly injected via their tail veins. Lung samples were collected at different time points, and their histology, gene expression profiles, and cell surface proteins were analyzed (see the online **Data Supplement** for details).

### 2.4 Statistical analysis

All values are expressed as means ± SD of three independent experiments. Data were analyzed using analysis of variance (ANOVA) followed by the Tukey–Kramer test. The statistical significance of differences between concentrations was set at *P < 0.05 or **P < 0.01, as indicated.

## 3 Results

### 3.1 Adhesion and activation of MSCs of different origins under ECM culture conditions

**Figure 1:**
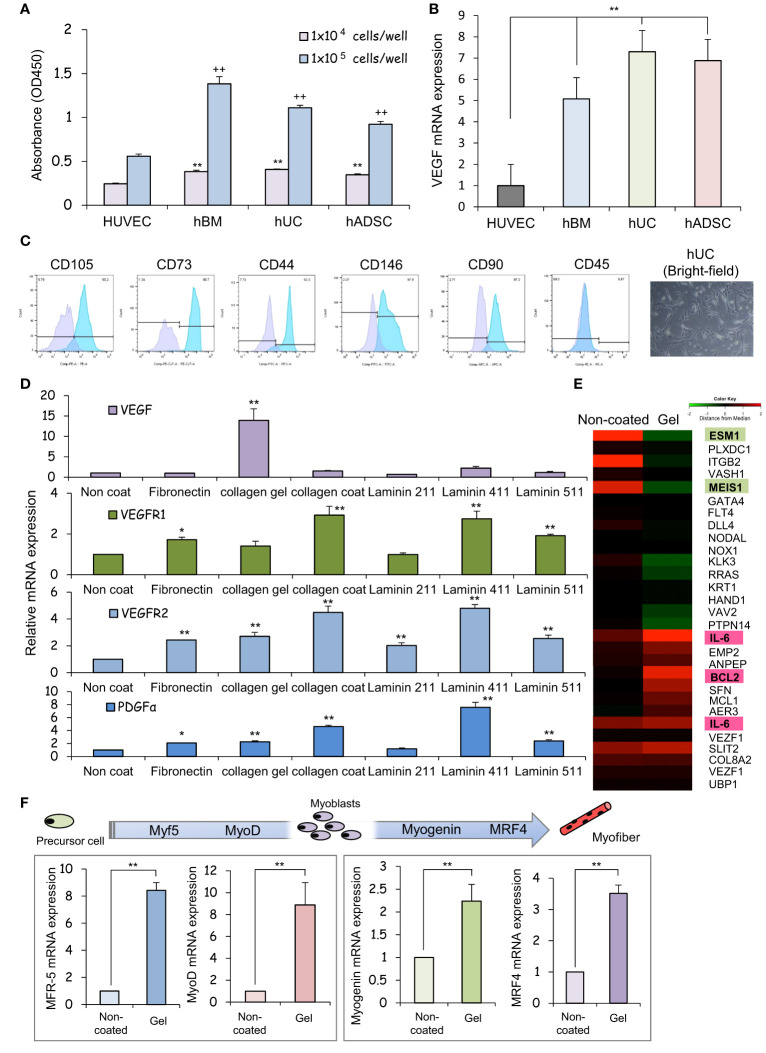
The human umbilical cord is a promising source of mesenchymal stem cells for COPD treatment. **(A)** Measurement of the viability of HUVECs and hBM-, hUC-, and hADSC-MSCs using CCK-8 assays, and comparison analysis of values at different cell densities. ** p < 0.01; ^++^ p < 0.01; compared to HUVEC on the same condition. **(B)** Relative mRNA expression of an angiogenic marker gene, VEGF, after culturing for 1 day; GAPDH was used as a control. ** p < 0.01 compared to HUVEC on the same condition. **(C)** Flow cytometry of the principal mesenchymal stem cell (MSC) markers. In each diagram, the name of the marker is indicated at the top, the fluorochrome used is indicated at the bottom, and the percentage of positive cells is indicated at the top right. The lower panel presents brightfield images of hUC-MSCs; DAPI was used to label cell nuclei. Scale bar: 20 μm **(D)** Relative mRNA expression levels of genes for angiogenic factors after culturing for 3 days; GAPDH was used as a control. * p < 0.05; ** p < 0.01; compared to hUC on Non coat culture condition. **(E)** hUC-MSCs were cultured on different ECM for 6 days; mRNAs were isolated and subjected to mRNA microarray analysis. In the mRNA heatmap, red and green indicate upregulated and downregulated mRNAs, respectively, in cells on type-I collagen gels. **(F)** Relative mRNA expression levels of genes encoding myogenic regulatory factors after culturing for 3 days; GAPDH was used as a control. ** p < 0.01 compared to hUC on Non coat culture condition.

After 24 hours of incubation, non-adherent cells were removed by washing with PBS. Cells that remained adherent to the well plate were considered as cells with adhesive ability, and the number of those cells was measured and evaluated using the cell counting kit-8 (CCK-8) assay. The results of CCK-8 assay showed that all types of MSCs exhibited greater attachment to plastic surfaces than human umbilical vein endothelial cells (HUVECs), especially at high cell seeding densities (**Figure 1A**). The ratio of VEGF expression to GAPDH gene expression in each MSCs was analyzed using HUVECs, which are known to promote cellular responses involved in angiogenesis, as a control. The mRNA expression of vascular endothelial growth factor (VEGF), was upregulated in each MSCs group in comparison to HUVEC (**Figure 1B**), indicating that the therapeutic potential of UC-MSCs is comparable to that of hBM-MSCs and hADSC-MSCs. MSCs can secrete the angiogenic factor VEGF, which promotes local angiogenesis, suggesting that MSC-based therapy after tissue injury may increase microvascular density and preserve organ function. For this VEGF gene expression analysis, HUVECs, hBM-MSCs, hADSC-MSCs, and hUC-MSCs were cells cultured on plastic surfaces to which no special coating such as ECM or stimulating factors were added.

### 3.2 Characterization of hUC cultures

The cells isolated from the Wharton’s jelly of the human umbilical cord exhibited a spindle-shaped fibroblast-like morphology and adhered well to the plastic (**Figure 1C**). These cells expressed stem cell-specific transcription factors such as OCT4, NANOG, and SOX2. Flow cytometry analysis revealed up- or downregulated expression of the following surface markers in the sorted cells: CD105-PE, 90.2%; CD73-PE-Cy7, 92.3%; CD146-FITC, 97.9%; CD90-PE, 97.3%, and 0.47% CD45-FITC. These results suggest that our cell cultures exhibited the typical MSC immunophenotype, i.e., CD105^+^/CD73^+^/CD146^+^/CD90^+^/CD45^-^ (**Figure 1C**).

### 3.3 Effect of ECM on the expression of vasculogenesis- and myoblast differentiation-related mRNAs in hUC-MSCs

We assessed the expression of neovascularization-related factors in hUC-MSCs cultured on different ECM proteins that constitute the basement membrane of alveolar epithelial cells. VEGF expression was higher in collagen I gel-coated wells than in laminin- or fibronectin-coated wells (**Figure 1D**). Coating with laminin 411, but not laminin 211 or laminin 511, promoted PDGFα expression. Since type I collagen gel cultures of hUC-MSCs exhibited vaso-inductive potential, cDNA microarray analysis was performed to evaluate hUC-MSCs’ effects on the regulation of vascular endothelium-related gene expression. A comparison of the microarray data obtained from cells cultured without collagen-1 coating (non-coated) and cells cultured on type I collagen gel revealed differences in the expression of VEGF induction-related genes (**Figure 1E**).

High levels of induction of vascular EC growth factors, IL-6, and BCL2 were found in cells cultured on type-I collagen gels. Additionally, the expression of genes such as endothelial cell-specific molecule 1 (*ESM1*) and meis homeobox 1 (*MEIS1*), which negatively regulate cell proliferation, was suppressed (**Figure 1E**). hUC-MSCs cultured on type I collagen gels also exhibited high expression of several genes involved in myofiber differentiation, indicating that these hUC-MSCs may heal injured areas of the airway smooth muscles (**Figure 1F**).

### 3.4 Effect of ECM combination on vascular endothelial cell markers

**Figure 2:**
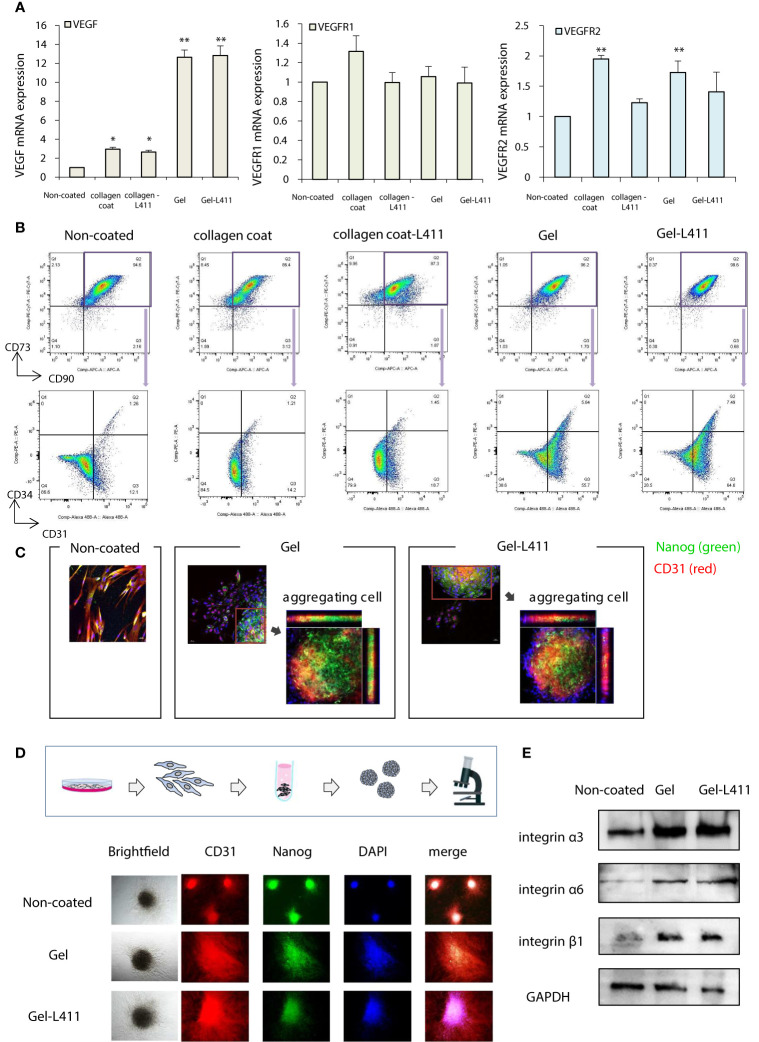
hUC-MSCs cultured on Gel and Gel-L411 induce angiogenesis. **(A)** hUC-MSCs were cultured on different ECMs for 6 days, and total RNA was isolated. Relative mRNA expression levels of genes encoding angiogenic factors; GAPDH was used as a control. **(B)** Flow cytometry gating strategy to quantify endothelial progenitor cells. Initially, the mesenchymal fraction was subgated at CD90 and CD73 (upper panel) and was further subgated into bivariate plots of CD31 and CD34 (lower panel). * p < 0.05; ** p < 0.01; compared to hUC on Non coat culture condition. **(C)** Gel and Gel-411 cells were exfoliated from the ECM, seeded into plates, and cultured for 3 days. Aggregation of these cells was observed in some wells, and the aggregated cells were Nanog (undifferentiated marker)-positive and CD31 (vascular induction marker)-positive. **(D)** hUC-MSCs removed from the ECM were cultured in 96-well concave microwells for 3 days and formed spheroids. **(E)** Optical images of hUC-MSC spheroids at different culture conditions and fluorescence microscopy images of CD31 and Nanog.

The additive effects of different combinations of ECMs on the mRNA, protein, surface marker, vasoinductive marker expression, and morphology of hUC-MSCs attached to these ECMs were evaluated. ECMs containing a collagen coating, collagen gel, and laminin 411 showed increased expression of vasoinductive markers (**Figure 1D**). Although cells cultured on collagen gels (Gel: type I collagen gel) and collagen gels containing mixed substrates (Gel-L411: type I collagen gel and laminin 411) showed greatly enhanced VEGF expression, laminin 411 did not exhibit an additive effect (**Figure 2A**).

Flow cytometry was used to characterize the differentiation and maturation processes. Changes in the expression of the cell surface markers, CD31 and CD34, were analyzed in the CD73^+^/CD90^+^ MSC populations (these are the most reliable vascular endothelial progenitor cell markers). High levels of CD31 were observed in cells cultured on Gel and Gel-L411 (approximately 61.4, 71.5, and 13.3% CD31^+^ cells grown in Gel, Gel-L411, and uncoated-cell culture conditions, respectively). The collagen-coated cultures did not show marked changes in the expression of these markers. The percentage of CD31^+^/CD34^+^ double-positive cells increased from 1.26% in uncoated cells to 5.64% in gel cells and 7.49% in Gel-L411 cells (**Figure 2B**), suggesting that vascular endothelial progenitor cell differentiation was induced in cultures grown in the presence of collagen gel, and cell differentiation was further enhanced by the combination of collagen gel and laminin 411. To observe the effects of ECM on cell morphology, hUC-MSCs were extracted from coated culture dishes, isolated by collagenase digestion, and seeded onto plastic slides. Although spindle-shaped fibroblast-like cells were observed in hUC-MSCs extracted from uncoated cells, hUC-MSCs extracted from Gel or Gel-L411 exhibited a heterogeneous morphology, including small round cells and spindle-shaped cells that showed spontaneous aggregation, suggesting that culturing on collagen gels induced cell-to-cell adhesion of hUC-MSCs. The aggregated cells comprised Nanog-positive undifferentiated MSCs and CD31^+^ vascular endothelial progenitor cells (**Figure 2C**).

Given these observations, we evaluated whether cell aggregation enhanced the angiogenic potential by inducing spheroid formation (**Figure 2D**). Spheroids formed from uncoated cultured cells exhibited regular, tightly packed aggregates, whereas cells cultured on Gel and Gel-L411 formed loose, irregularly shaped aggregates. Regardless of the culture conditions, none of the hUC-MSC spheroids were positive for Nanog or CD31, indicating that the aggregation of undifferentiated hUC-MSCs promoted differentiation and angiogenesis. Western blot analysis revealed that the expression of the cell adhesion molecule integrin was markedly upregulated in cells cultured on Gel and Gel-L411; however, there was no observable difference in integrin expression in hUC-MSCs cultured in the presence or absence of laminin 411 (**Figure 2E**).

### 3.5 *In vitro* effects of hUC-MSCs in lung injury models

**Figure 3:**
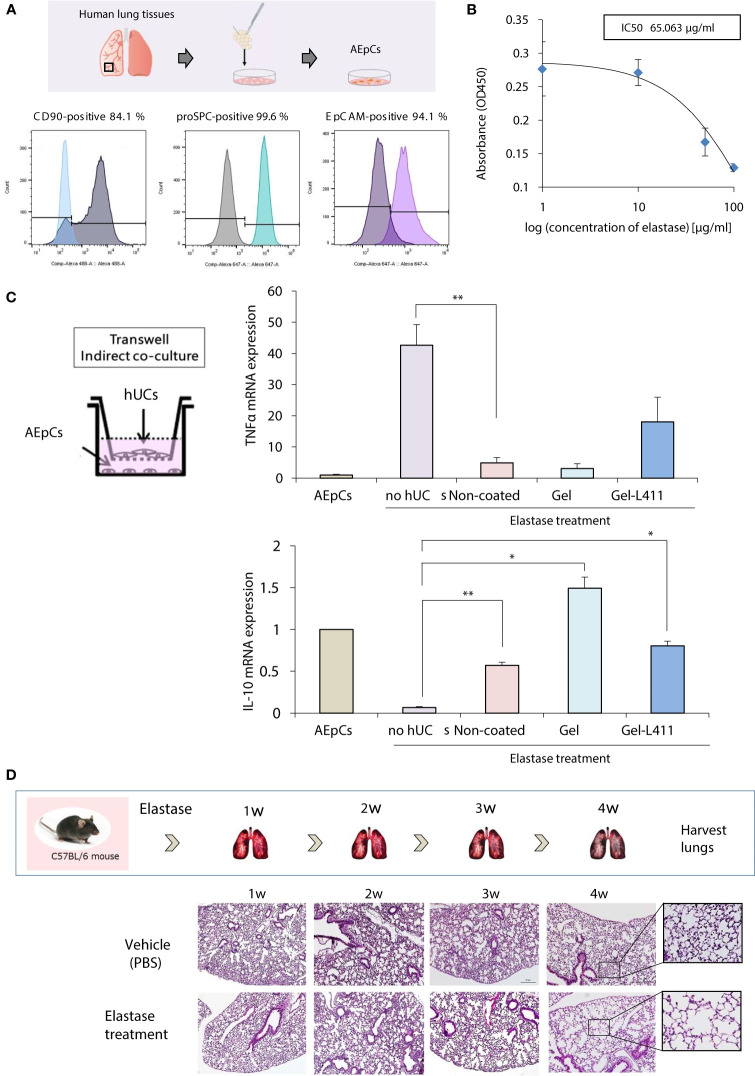
*In vitro* models of epithelial–mesenchymal crosstalk in the lung and establishment of COPD mouse models. **(A)** Flow cytometry of the principal mesenchymal stem cell (MSC) markers. In each diagram, the name of the marker is indicated at the top, the fluorochrome used is indicated at the bottom, and the percentage of positive cells is indicated at the top right. **(B)** Dose-response curves of IC_50_ for elastase: AEpCs cells were treated for 24 h with 0, 1, 10, 50 and 100 μM of elastase. **(C)** Establishment of the AEpC-hUC-MSC co-culture system. The transwell co-culture systems were established in 6-well plates. To assess the effect of co-culture system on AEpCs, we used AEpCs as a control group. Inflammation was evaluated using TNFα and anti-inflammatory effects using IL-10. * p < 0.05; ** p < 0.01; compared to AEpCs on Elastase treatment culture condition. **(D)** Induction of a COPD-like phenotype in C57BL/6 wild-type mice induced with elastase exposure: representative weekly histopathology based on single intranasal administration of elastase for 4 weeks (n = 5 per group); scale bar: 200 μm.

Alveolar epithelial progenitor cells (AEpCs) were isolated from adult human lung tissues for *in vitro* analysis (**Figure 3A**). These progenitor cells express the MSC surface marker CD90 and factors associated with alveolar type II cells, such as the epithelial cell adhesion molecule (EpCAM) and pro-surfactant protein C (pro-SPC). The half-maximal inhibitory concentration (IC_50_) value of elastase against these AEpCs was 65.063 μg/ml (**Figure 3B**). To evaluate the effect of UC-MSC signaling on the alveolar epithelium, hUC-MSCs and AEpCs were indirectly co-cultured on Transwell^®^ membranes (**Figure 3C**). qRT-PCR showed significant inhibition of expression of the inflammatory marker, tumor necrosis factor (TNF)-α, and upregulation of expression of the anti-inflammatory marker, interleukin (IL)-10, in hUC-MSCs that were co-cultured with AEpCs.

### 3.6 hUC-MSCs cultured on ECM promote lung regeneration and macrophage polarization after elastase treatment *in vivo*

A preclinical COPD model was used to determine the *in vivo* effects of human MSC therapy. The COPD model was established by intranasal administration of elastase to mice over 4 weeks to induce emphysema-like changes in the lungs (**Figure 3D**).

**Figure 4:**
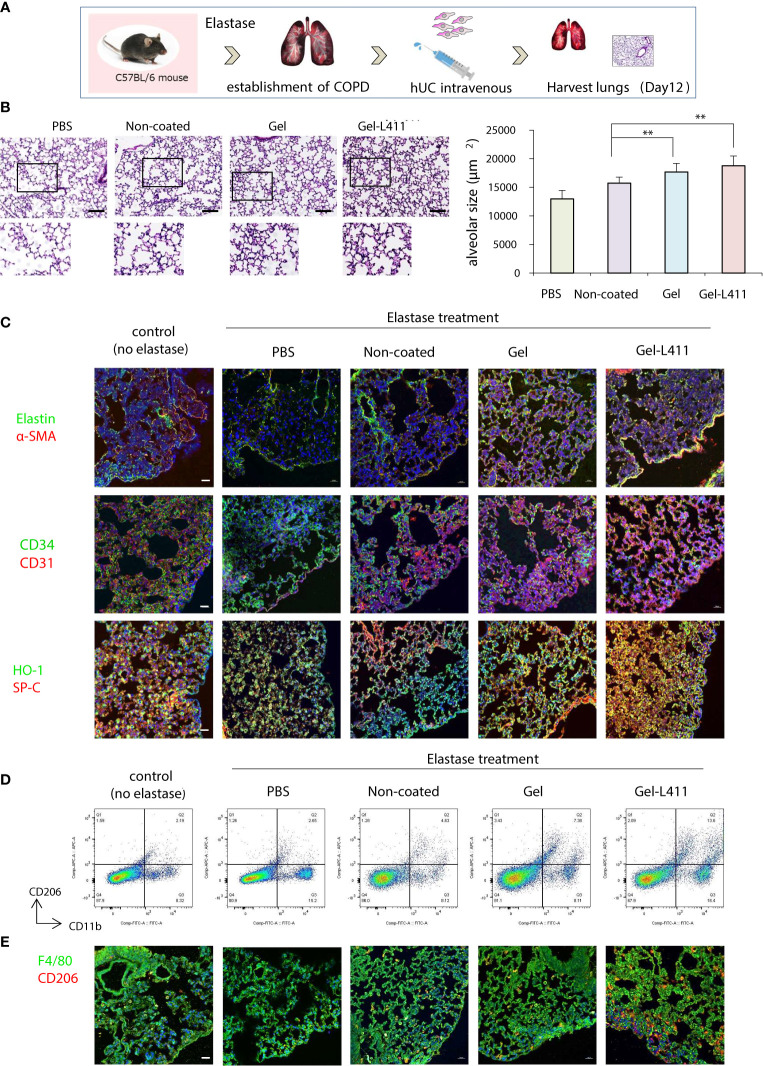
Modified hUC-MSCs exert therapeutic effects on lung tissue in a mouse model of COPD. **(A)** Schematic representation of the COPD model based on cell administration after intranasal administration of elastase. **(B)** Histopathological evaluation in the lung tissue of elastase-induced mice after 12 days of treatment (n = 5 per group): H&E staining, scale bar: 200 μm. ** p < 0.01 compared to hUC injection group on Non coat culture condition. **(C)** Detection of Elastin, α-SMA, CD34, CD31 HO-1, and proSPC-positive cells in UC-MSCs using indirect immunofluorescence and a confocal laser scanning system (n = 5 per group). Magnification, 400×; scale bar: 20 μm. **(D)** Flow cytometry gating strategy to quantify macrophage cell populations (n = 5 per group). **(E)** Representative images of immunofluorescence staining of F4/80 (green) and CD206 (red) in hUC-MSCs (n = 5 per group). Magnification 400×.

Four weeks after elastase administration, hUC-MSCs were administered to the mice, and the lungs were harvested on day 12 for analysis (**Figure 4A**). Alveolar damage was assessed using hematoxylin and eosin (H&E) staining. The total surface area of the alveoli increased after administration of hUC-MSCs, with a significant tissue repair effect, especially in the Gel-L411 cultured cell group (**Figure 4B**). The localization of alpha-smooth muscle actin (α-SMA)-positive cells was examined by immunostaining to confirm the contribution of hUC-MSCs in the formation of vascular smooth muscle cells along the small airways (bronchioles) and alveolar walls of the lungs. Immunofluorescence analysis of the elastase-treated lungs showed that the expression of elastin, a major component of the connective tissue that organizes the alveoli, was suppressed, whereas that of α-SMA increased after hUC-MSC injection (**Figure 4C**). The expression of CD31, also known as platelet/endothelial cell adhesion molecule-1 (PECAM-1, an adhesion molecule that accumulates at adhesion sites between vascular endothelial cells and increases integrin binding activity), was markedly upregulated in the Gel and Gel-L411 cell groups. The expression of the hematopoietic progenitor cell marker, CD34, did not markedly increase (**Figure 4C**). The antioxidant protein heme oxygenase-1 (HO-1) was detected mainly in alveolar type II cells, indicating the co-localization of HO-1 with proSPC (**Figure 4C**).

Alveolar macrophages may play an important role in the pathogenesis of COPD because they express proteases such as matrix metalloproteinases (MMPs) and cathepsins (21). Therefore, we examined the polarity of macrophages and analyzed the mechanisms underlying alveolar regeneration. hUC-MSCs were co-immunolabeled with CD11b, a monocyte marker, and CD206, an M2 anti-inflammatory macrophage marker, to investigate the phenotypic changes that occur upon the administration of hUC-MSCs to macrophages present in lung tissues. Flow cytometric analysis of isolated lung monocytes revealed an overall increase in CD11b levels in all elastase-treated groups. The hUC injection, especially the injection of Gel-L411 cells, resulted in an increased number of M2 macrophages (**Figure 4D**).

To determine whether the injected hUC-MSCs interacted with macrophages, lungs collected on day 12 were immunostained with antibodies against F4/80 (a pan-macrophage marker) and CD206 (**Figure 4E**). In line with the fluorescence-activated cell sorting (FACS) analysis, we confirmed that the overall polarization of macrophages in lung tissues shifted toward an M2-predominant type after injection of Gel-L411 cells.

### 3.7 Therapeutic efficacy of administered hUC-MSCs in COPD-related osteoporosis

**Figure 5:**
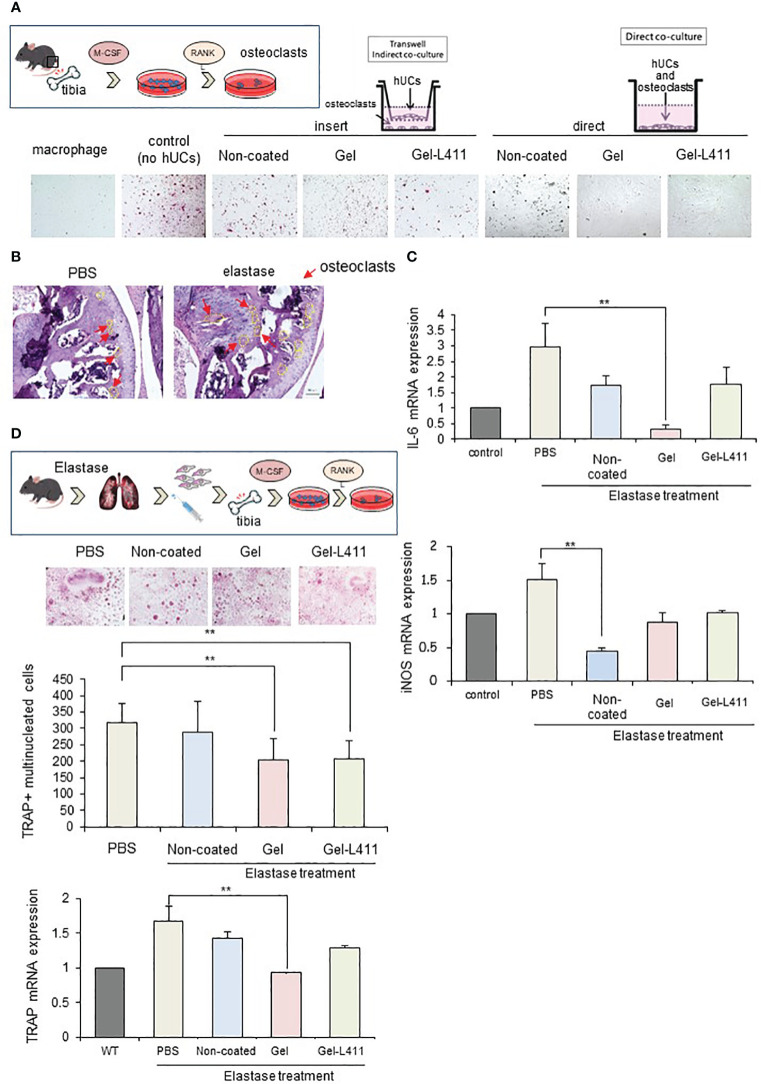
Modified hUC-MSCs exert therapeutic effects on osteoporosis in a mouse model of COPD. **(A)** Schematic of osteoclast induction from the bone marrow extracted from wild-type mice. To examine the interaction between osteoclast differentiation and modified hUC-MSCs, we established co-culture systems. The left group is a transwell-based non-contact culture, and the right group is a direct culture. Purified TRAP-positive osteoclasts formed in cocultures of hUC-MSCs and bone marrow cells. **(B)** Schematic of osteoclast induction from the bone marrow extracted from a mouse model of COPD after cell injection. TRAP-positive osteoclast formation induced after bone marrow extraction from mice treated with hUC-MSCs injections. Quantitative analysis showing the number of osteoclasts per well and TRAP mRNA expression. (n = 5 per group) **(C)** Characterization of gene expression of the inflammatory markers interleukin-6 (IL-6) and inducible nitric oxide synthase (iNOS). ** p < 0.01 compared to hUC injection group on Non coat culture condition. **(D)** TRAP staining and increased osteoclast formation in the femoral head in COPD-induced osteoporosis. mRNAexpression levels of TRAP were determined by qPCR.

We examined whether Gel- and Gel-L411-cultured cells, when co-cultured with receptor activator of NF-κB ligand (RANKL)-induced osteoclast progenitors and hUC-MSCs, affected osteoclastogenesis *in vitro*. Bone marrow from wild-type mice was extracted, seeded in well plates, and cultured in the presence of M-CSF and RANKL to differentiate into mature multinucleated tartrate-resistant acid phosphatase (TRAP)-positive osteoclasts. The contact approach between osteoclasts and hUC-MSCs was evaluated using a non-contact co-culture model of osteoclasts and hUC-MSCs using Transwell (left side of **Figure 5A**; insert group) and a direct co-culture model in which hUC-MSCs were added directly to osteoclasts (right side of **Figure 5A**; direct group). The group of bone marrow cells to which only M-CSF was added was designated “macrophages” and the group to which M-CSF and RANKL were added was designated “control (no hUCs)”. The hUCs added to osteoclasts in both the insert and direct groups were hUC-MSCs cultured without coating and “non-coated”, “gel” on gel and “Gel-411” on gel with Laminin 411.

TRAP staining (red) showed that osteoclastogenesis was almost completely inhibited in the Direct assay, indicating that cell-to-cell contact stimulated osteoclastogenesis, which occurs in the presence of soluble mediators (**Figure 5A**).

We also evaluated the morphology and number of osteoclast progenitor cells by culturing bone marrow cells (obtained from the tibiae of the control and experimental mice) in the presence of RANKL and M-CSF for five days. Numerous TRAP-positive multinucleated osteoclasts were observed in the control group. The number of osteoclasts and expression of the mature osteoclast marker, TRAP, in cultures of COPD mouse models were significantly lower in the Gel and Gel-L411 groups than in the control group (**Figure 5B**). Expression of the M1 markers, IL-6 and inducible nitric oxide synthase (iNOS), in bone marrow cells (isolated from experimental mice) decreased in the hUC-MSC-treated group and was suppressed to a greater extent in the Gel group than in the Gel-L411 group (**Figures 5C, D**).

### 3.8 Gel and Gel-L411 interact with macrophages in the mouse lung after infusion

**Figure 6:**
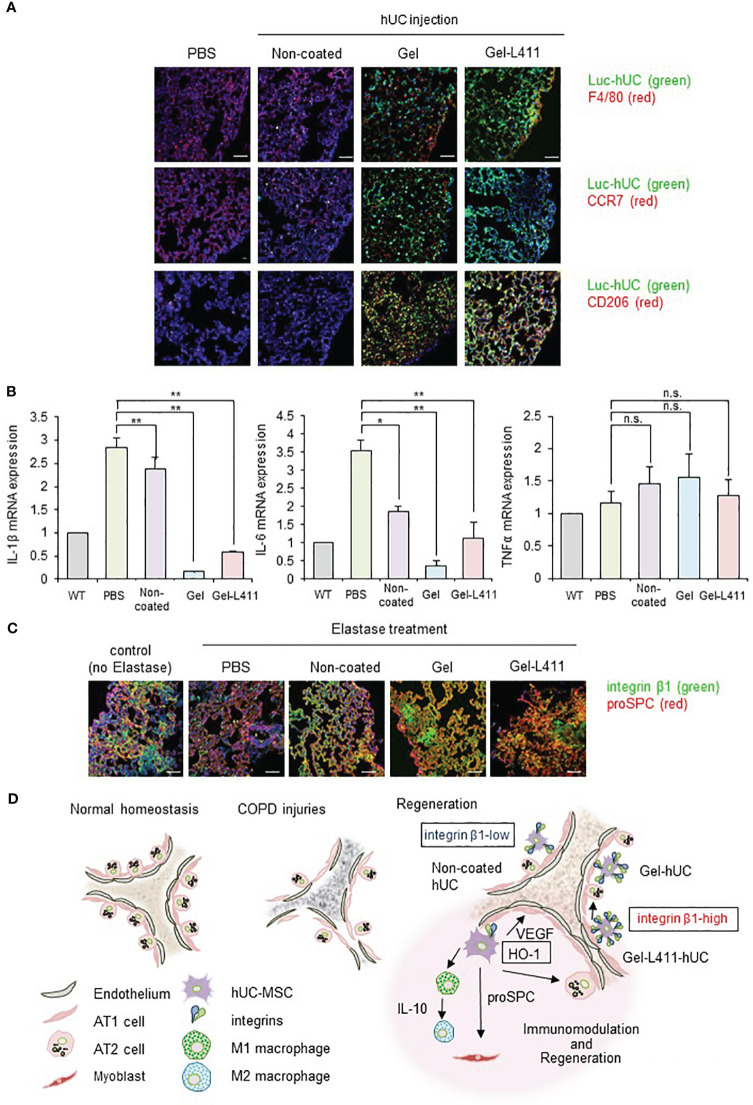
dUC-MSCs promote epithelial spreading and lung regeneration **(A)** Luciferase-labeled hUC-MSCs were injected, and their biological kinetics were analyzed using fluorescence imaging. Immunostained images of lung tissue collected after 24 h of cell injection into COPD model mice: Luc-hUC (green) and F4/80 (red). Scale bar: 40 μm **(B)** Gene expression of inflammation markers in RNA extracted from lung tissue 24 h after hUC-MSC injection * p < 0.05, ** p < 0.01, n.s. = Not significant; compared to hUC injection group on Non coat culture condition. **(C)** Immunostained image of lung tissue using confocal laser microscopy (12 days after cell injection): integrin β1 (green) and proSPC (red). Scale bar: 40 μm. **(D)** Regeneration of the alveolar epithelium after injury. Elastase-mediated injury results in extensive destruction of all alveolar epithelial cells. Surviving ATII cells are activated and proliferate after injury, restoring the alveolar epithelium. A progenitor cell population that expresses high levels of integrin β1 appears to contribute to alveolar epithelial regeneration.

To investigate the function of hUC-MSCs *in vivo*, we analyzed their behavior using luciferase-expressing cells (Luc-hUCs). hUC-MSCs almost disappeared 24 h after intravenous administration of Luc-hUCs. Several Luc-hUCs cultured on the Gel or Gel-L411 were observed in the lung samples (**Figure 6A**). To determine whether hUC-MSCs interacted with macrophages *in vivo* after injection, lung samples were immunolabeled with antibodies against the macrophage marker F4/80. In the Gel and Gel-L411 groups, hUC-MSCs were distributed throughout the lung tissue, accumulated on the outer margins, and co-localized with macrophages. Injection of hUC-MSCs induced the generation of CD206^+^ M2-type macrophages, while CCR7^+^ M1-type macrophages were not formed, suggesting that the injection of hUC-MSCs induced an M2 polarization shift in macrophages, resulting in anti-inflammatory effects.

A noticeable change in TNFα expression was not observed in the lungs 24 h after cell administration (**Figure 6B**) but those of IL-1β and IL-6 were suppressed, indicating that cells cultured on the ECM induced anti-inflammatory effects in the lungs.

### 3.9 Laminin-411 promotes epithelial spreading

Integrin β1 co-localized with proSPC in the COPD mouse models injected with Gel or Gel-L411 cells (**Figure 6C**). Considering that attached hUC-MSCs are phagocytosed by macrophages, *in vitro* experiments (**Figures 2C–E**) suggested that integrin β1 may be the key factor that induced the adhesion of hUC-MSCs, leading to alveolus formation in the COPD model. This result is agreement with previously reports that human mesenchymal stem cells cultured on collagen gels differentiate into epithelial cells through the formation of cytokeratin-18 (22).

## 4 Discussion

In this study, we developed dUC-MSCs by culturing UC-MSCs on a type I collagen gel and laminin 411. dUC-MSCs were injected via the tail vein of COPD mice, where they were attached to the lung tissue and survived for a long duration. Mice injected with dUC-MSCs showed a greater alleviation of elastase-induced COPD symptoms than those injected with hUC-MSCs. Our experimental data suggest that tissue regeneration occurs because of the binding of dUC-MSCs to the ECM through integrin-mediated cell adhesion mechanisms (23–26). Thus, dUC-MSCs promoted macrophage differentiation through cytokine secretion or direct contact. To the best of our knowledge, this is the first report on the role of modified cells in stimulating lung epithelial cell induction and lung tissue regeneration for COPD treatment. Notably, gene modification was not required to strengthen MSCs. Overall, we developed novel designer cells that may be involved in anti-inflammatory, homeostatic, injury repair, and disease resistance processes.

Research in this field is typically conducted using BM-MSCs or AD-MSCs. MSCs obtained from other sources, such as the umbilical cord and placenta, are also used for therapeutic angiogenesis (27). Perinatal stem cells (hUC-MSCs) exhibit pluripotency, multipotent tissue maintenance, a high degree of plasticity, and immunomodulatory activity and lack tumorigenicity; they are considered the best sources of allogeneic xenografts (28–30). Furthermore, UC-MSCs secreted VEGFs and uniformly expressed endothelial markers without altering their cellular organization or morphology (30). We examined VEGF expression in MSCs derived from various tissues and confirmed that UC-MSCs exhibited enhanced VEGF production (31). hUC-MSCs are a promising cell source and have many advantages over BM-MSCs and AD-MSCs.

hUC-MSCs cultured on collagen I and laminin 411 (Gel-L411 dUC-MSCs) showed the highest expression levels of vascular endothelial markers and myofibroblast progenitor cell markers *in vitro* and retained their anti-inflammatory effects without undergoing genetic modifications. During the regeneration of the destroyed basement membrane and ECM, MSCs differentiate into myofibroblasts (32) and pulmonary capillary endothelial cells (33), and their interaction contributes to angiogenesis and alveolus formation (**Figure 4C**). dUC-MSCs exhibited a higher adhesion capacity than normally cultured hUC-MSCs. ECM interactions and their processes in tissue repair are mediated by integrins, generally through the intracellular signaling molecules, Rho and Rac, which also have synergistic effects on differentiation into the epithelial lineage (34–38). Integrin β1 activity was upregulated in both Gel- and Gel-L411-dUC-MSCs, demonstrating the positive and significant effects of these substrates (**Figure 6D**).

Furthermore, MSCs contribute to changing the phenotype of macrophages from M1 (inflammatory) to M2 (anti-inflammatory). M2 macrophages play important roles in regulating inflammation, angiogenesis, debris removal, and tissue remodeling (21, 39–42). This was confirmed by flow cytometry (**Figure 4D**), which showed a polarity change toward the M2 macrophage state (along with an increase in macrophage number) in the dUC-treated group, suggesting the involvement of M2 macrophages in tissue repair.

In cell injection experiments using COPD mouse models, dUC-MSCs adhered for a long time, whereas hUC-MSCs adhered for shorter periods in normal cultures. The primary issue in using therapeutic cells to treat lung diseases is the failure of the cells to attach to the target area. In previous clinical trials, two doses of MSCs were administered in one cycle for at least two cycles (four doses in total) (ClinicalTrials.gov; No.: NCT00683722; URL: www.clinicaltrials.gov). In our study, dUC-MSCs survived for a longer period than normally cultured UC-MSCs in COPD mouse models. Therefore, the use of dUCs may reduce the number of times these therapeutic cells need to be administered.

Systemic inflammation associated with COPD can contribute to the pathogenesis of osteoporosis. Key inflammatory cytokines, such as iNOS and IL-6, interact with RANKL (43). dUC-MSCs inhibit the formation of abnormal osteoclasts by suppressing the expression of inflammatory mediators. Therefore, dUC-MSCs can be used to treat osteoporosis and other comorbidities caused by inflammatory reactions.

Integrin β1 expression in the lung epithelium is required for airway branching morphogenesis, alveolus formation, and homeostasis (44). Integrin β1 regulates epithelial cell adhesion and migration, alveolar cell differentiation, and ECM deposition in the alveolar septum (45). Integrin β1 promoted angiogenesis by stimulating smooth muscle formation by modulating the production of reactive oxygen species (**Figures 4C**, **6C**). This appears to be a novel mechanism by which cell–ECM interactions modulate lung inflammation and alveolar septum formation.

However, while culture with gel-form type I collagen activates signal transduction that regulates the induction of differentiation and other factors, it also induces growth suppression. Therefore, for clinical applications, it is necessary to establish a culture environment that allows for mass production while maintaining cellular characteristics; further research is required to confirm this hypothesis. Moreover, dUC-MSCs are not suitable for practical use because they are cultured on gels and require collagenase treatment for cell harvesting. Therefore, we are researching peptides that can mimic collagen-gel culture to secure cell numbers in normal planar culture; accordingly, we have commenced an attempt to mass-produce therapeutic cells with high tissue-repair capacity.

In summary, in this study, we developed therapeutic cells to overcome the nonviability of transplanted cells during cell transplantation therapy. The adhesion properties of dUC-MSCs were modified by binding to cell adhesion molecules, allowing the cells to maintain their adhesive function even after detachment. We focused on elucidating the mechanisms underlying the immune regulation mediated by UC-MSCs and aimed to establish a culture method optimized for the induction of endogenous signaling pathways that could alter cell behavior. This innovative cell therapy method can enable the large-scale production of cell products for transplantation without using any genetic manipulation strategies.

## Data availability statement

The original contributions presented in the study are included in the article/**Supplementary Material**, further inquiries can be directed to the corresponding author/s.

## Ethics statement

The studies involving humans were approved by Nagasaki University Research Ethics Committee No.22122301. The studies were conducted in accordance with the local legislation and institutional requirements. The participants provided their written informed consent to participate in this study. The animal study was approved by the Nagasaki University Institutional Animal Care and Use Committee guidelines and the ethics committee of Nagasaki University (Approval Number: 2202281776). The study was conducted in accordance with the local legislation and institutional requirements.

## Author contributions

MIw: Writing – original draft, Writing – review & editing, Funding acquisition. TN-I: Formal analysis, Writing – review & editing. RD: Funding acquisition, Writing – review & editing. YT: Funding acquisition, Methodology, Writing – review & editing. MIs: Formal analysis, Funding acquisition, Methodology, Writing – review & editing. HY: Methodology, Writing – review & editing. KM: Writing – review & editing. KT: Writing – review & editing. TN: Writing – review & editing. TT: Funding acquisition, Writing – review & editing.
