# Supplementary material for: Designer umbilical cord-stem cells induce alveolar wall regeneration in pulmonary disease models
Source: Front Immunol. 2024 Apr 30;15:1384718. doi: 10.3389/fimmu.2024.1384718 (PMC11091323; doi:10.3389/fimmu.2024.1384718)
Supplement: Supplementary file 1 [file Table_1.docx]

**Designer umbilical cord-stem cells induce alveolar wall regeneration in pulmonary disease models**

Mayumi Iwatake, Tokiko Nagamura-Inoue, Ryoichiro Doi, Yukinori Tanoue, Mitsutoshi Ishii, Hiroshi Yukawa, and Tomoshi Tsuchiya

ONLINE DATA SUPPLEMENT

**Supplementary materials and methods**

**Cell culture**

Human umbilical cord mesenchymal stem cells (UC-MSCs (RRID:CVCL_1D58)) were provided by Dr. Nagamura (Tokyo University, Japan). Low-glucose Dulbecco's modified Eagle's medium (DMEM) with L-glutamine) was procured from Sigma-Aldrich (Cat D6046-500ML, St. Louis, MO.). Fetal bovine serum (FBS) was obtained from Nichirei Bioscience Inc. The cells were cultured in low-glucose DMEM supplemented with 10% FBS and 100 U/ml penicillin and streptomycin under mycoplasma-free conditions at 37˚C in 5% CO_2_. Human bone marrow mesenchymal stem cells (BM-MSCs; HMSC.BM-100) were purchased from Cellular Engineering Technologies (Coralville, IA, USA). Cells were passaged by treatment with 0.25% trypsin/EDTA (Cat 201-16945, WAKO Pure Chemical Industries, Osaka, Japan) in phosphate-buffered saline (PBS) and used between passages 4 and 6.

Six-well culture plates (Nunc Cell-Culture Treated Multidishes, 140685) were used for culture. The collagen gel used was Cellmatrix Type I-A (collagen concentration: 3 mg/mL [pH 3]) from Nitta Gelatin Co (Osaka, Japan). According to the manufacturer’s protocol (https://www.nitta-gelatin.co.jp/ja/product/biomedical/Cellmatrix/cellmatrix.html), Cellmatrix Type I-A (A), 10-fold concentrated culture medium (Nitta Gelatin) (B), and buffer solution for reconstitution (Cat 637-00653, Nitta Gelatin, Osaka, Japan) (C) were prepared; A (8 volumes) and B (1 volume) were mixed well without foaming while cooling, and then, C (1 volume) was added and mixed. The resulting collagen solution with a collagen concentration of 2.4 mg/mL was gelatinized by adding 2 mL per well and heating at 37°C for 30 minutes (the gel thickness was approximately 2 mm).

　The collagen used for coating was Nitta Gelatin's Cellmatrix Type I-C (collagen concentration: 3 mg/mL [pH 3]); Cellmatrix Type I-C was diluted by adding 10x hydrochloric acid solution (pH 3). The resulting collagen solution with a collagen concentration of 0.3 mg/mL was coated by adding 1 mL to each well and being allowed to stand for 1 h at room temperature (20°C to 25°C).

　Fibronectin solution (human) (PromoCell, C-43060) was diluted to 50 μg/mL, added to wells to achieve a coating concentration of 5 μg/cm^2^, and allowed to stand for 1 h at room temperature for coating. Laminin 211E8, laminin 411E8, and laminin 511E8 were iMatrix-211, iMatrix-411 and iMatrix-511 (all from Nippi, 0.5 mg/mL), respectively. They were coated to a coating concentration of 0.5 μg/cm^2^ according to the product instruction manual. Uncoated plates were used as controls.

**Separation of human lung cells**

For progenitor cell isolation, human lung tissue was obtained from patients with lung tumors, from a site away from the tumor. The lung cells were bluntly separated from the pleura, and the lung specimens were cut into 1 x 1 x 1 cm^3^ pieces. The cells were treated with DNase. After washing, they were cultured to obtain alveolar progenitor cells.

**Ethical compliance**

All mouse maintenance and procedures were conducted under the guidance of the Nagasaki University Animal Center Institutional Animal Care and Use Committee in accordance with the institutional and regulatory guidelines (Approval No. 1807101460-2). All human lung experiments were approved by the Nagasaki University Hospital Clinical Research Ethics Committee (license No. 19070810-4) and were in accordance with the guidelines outlined by the Board at Nagasaki University Hospital. Written informed consent was obtained from each subject.

**Animal model of COPD**

Eight- to ten-week-old male C57BL/6 (B6) mice (n = 5 per group) were purchased from CLEA Japan, Inc. (Tokyo, Japan). All mice were maintained under clean conventional conditions (temperature of the breeding room: 20～26℃, light/dark hours: 150-300 lux at 40-85 cm, water: for water bottles, feeding: continuous feeding method) at the Nagasaki University animal center.

For emphysema induction, the animals were anesthetized with isoflurane and challenged with an intranasal instillation of 50 µL of porcine pancreatic elastase (PPE) (058-05361; FUJIFILM Wako Pure Chemical Corporation, Japan). The animals received one dose only on day 0. Control animals received 50 µL of 0.9% saline solution (vehicle). Lung samples were fixed with 10% neutral buffered formalin (Kanto Chemical Co., Inc., Japan), paraffin embedded, sliced at 5-μm thickness, and stained with hematoxylin and eosin (H&E) for histological analysis.

**Cell Counting Kit-8 (CCK-8) assay**

To examine grafted cell survival, cell viability and proliferation were determined using a CCK-8 assay. After cells were cultured for 24 h, the culture medium was replaced with a serum-free culture medium containing CCK-8 (0.5 mg/ml). After 1 h of culture, absorbance was measured at 450 nm using Multiskan FC microplate reader (Thermo Fisher Scientific K.K., Tokyo, Japan). Optical density (OD) at 450 nm was proportional to the number of cells.

IC_50_ values are defined as the 50% cell growth inhibitory concentrations of each compound, which are obtained from the following equation: IC_50_ = 10^[LOG(A/B)x(50-C)/(D-C) + LOG(B)], where A is the higher concentration of two values that sandwich IC_50_, B is the lower concentration of two values that sandwich IC_50_, C is cell viability (%) at B, and D is cell viability (%) at A.

**Flow cytometry**

After the third passage, UC-MSCs were trypsinized (0.25% trypsin EDTA), washed twice with PBS, and stained on ice with monoclonal antibodies against CD105-PerCP, CD146-CFS, CD90-APC, and CD45-PE (Human Mesenchymal Stem Cell Flow Kit, R&D Systems) and CD73-APC-Cy7 (BioLegend 344009). PE-IgG1 and FITC-IgG1 were used as isotype controls. The stained cells were analyzed by flow cytometry (FACS Lyric; Becton-Dickinson, San Jose, CA, USA) and FlowJo analysis software (Becton, Dickinson, and Co.).

The following primary conjugated antibodies were used in flow cytometry experiments: CD11b–FITC (BioLegend 101206), CD206-APC-Cy7 (BioLegend 321120), CD34–PE (eBioscience 12-0349-42), and CD31-Alexa488 (BioLegend 303109).

**Two-dimensional collagen culture system**

The two-dimensional collagen gel culture system was prepared according to the manufacturer’s protocol. Briefly, Cellmatrix Type I-A porcine tendon collagen (Component A; Nitta Gelatin, Inc., Osaka, Japan) was mixed with 10× Ham's F12 (Component B) and a reconstruction buffer (50 mM NaHCO_3_ and 200 mM HEPES in 100 ml 0.05 N NaOH; Component C) in an 8:1:1 (A:B:C) volume ratio. A total of 2 ml collagen gel solution containing the cells was poured into a 60-mm plastic Petri dish (Falcon Plastics, Oxnard, CA), and the culture dish was immediately warmed to 37 ˚C to allow a gel to form. After 30 minutes, when the gel was firm enough, UC-MSCs were seeded onto gels at 2.5 × 10^5^ cells.

**Separation of human lung cells**

For cell isolation, human lung tissue was obtained from patients with lung tumors who underwent lung resection at the Department of Surgical Oncology, Nagasaki University Hospital. All subjects gave their informed consent. Lung tissue was obtained from a site away from the tumor. Patients’ characteristics are shown in Table E2. Human lung cells were bluntly separated from the pleura, and lung specimens were cut into 1 x 1 x 1 cm^3^ pieces. The samples were incubated with collagenase (17100017, Thermo Fisher Scientific), dispase (354235, Corning®), and DNase (10104159001, Thermo Fisher Scientific) for 1 h at 37°C with shaking. Enzymatically digested samples were filtered through a 100-µm cell strainer. After treatment with erythrocyte lysis buffer (Roche Applied Science), they were resuspended in DMEM/F12 (Invitrogen, Carlsbad, CA, USA) containing 10% FBS, 1% amino acid solution (Invitrogen), 100 units/ml penicillin, 100 μg/ml streptomycin (antibiotics; Sigma-Aldrich), and 2.5 μg/ml amphotericin B.

***In vitro* co-culture model**

Co-culture of hUC-MSCs and AEpCs or osteoclasts was performed using a transwell system (BD Biosciences).

First, 1 x 10^4^ AEpCs were seeded on inverted transwells, left for 2 h to attach, and then placed in 12-well companion plates. After 24 h, 0.4 x 10^4^ hUC-MSCs were seeded in new companion plates and were combined with the AEpC-populated transwells. Only AEpCs were seeded as controls. The whole co-culture was performed in low-glucose DMEM supplemented with 10% FBS and 100 U/ml penicillin and streptomycin under mycoplasma-free conditions at 37˚C in 5% CO_2_.

**Western blot analysis**

Cells were rinsed twice with ice-cold PBS and lysed in a cell lysis buffer (50 mM Tris-HCl [pH 8.0], 1% Nonidet P-40, 0.5% sodium deoxycholate, 0.1% SDS, 150 mM NaCl, 1 mM PMSF, and proteinase inhibitor cocktail). The protein concentration of each sample was measured using BCA Protein Assay Reagent (Thermo Pierce, Rockford, IL, USA). Lysate proteins (5 µg) were resolved in an SDS-PAGE gel and electroblotted onto a polyvinylidene difluoride membrane. The blots were blocked with 3% milk/TBST for 1 h at room temperature, probed with various primary Abs overnight at 4°C, washed three times with PBS, incubated with horseradish peroxidase-conjugated secondary Abs (anti-rabbit IgG, 1:2000; and anti-mouse IgG, 1:2000; Cell Signaling Technology) for 1 hour at room temperature, and finally detected with ECL-Prime (GE Healthcare Life Sciences, Tokyo, Japan). The immunoreactive bands were analyzed using an LAS-4000mini (Fujifilm, Tokyo, Japan).

**Quantitative polymerase chain reaction analysis**

Total RNA was extracted using TRIzol Reagent (Invitrogen). Reverse transcription was performed using a ReverTra Ace qPCR RT Kit (Toyobo, Shiga, Japan). Quantitative real-time PCR was performed using an AriaMx Real-time PCR System (Agilent Technologies, La Jolla, CA, USA). The cDNA was amplified using Brilliant III Ultra-Fast SYBR QPCR Master Mix (Agilent), according to the manufacturer’s instructions. The primer sequences are described in Table E1. The qRT-PCR assays were performed in duplicate in three independent experiments for each experimental condition. Rat glyceraldehyde-3-phosphate dehydrogenase (GAPDH) was used for normalization of the qRT-PCR results.

**Immunofluorescence and histology**

UC-MSCs were fixed with 4% paraformaldehyde at 4°C for 30 min, permeabilized with 0.01% digitonin for 10 min, and blocked with 5% normal goat serum, followed by incubation with primary antibodies diluted with 5% normal goat serum and incubation overnight at 4°C. After three washes in PBS, cells were incubated with primary antibodies (1:100) (Nanog (NL1997G, R&D systems), CD31 (LS-C348736, LifeSpan BioSciences), VEGF (ab46154, Abcam) and CD146 (FAB932F, R&D systems) in blocking solution for 1 h at room temperature.

Paraffin sections were stained with H&E (Sigma) or immunostained. For immunofluorescence, after the deparaffinization and antigen retrieval process, sections were incubated with primary antibodies according to the manufacturer’s instructions after blocking for 1 h at room temperature. The slides were then washed and incubated with the appropriate secondary antibodies and labeling dyes. Secondary antibodies were coupled to Alexa-488 or Alexa-568 fluorochromes. After washing, tissue sections were mounted with VECTASHIELD Mounting Medium (Vector Laboratories). Primary antibodies used were: F4/80 (MCA497GA, Bio-Rad), CD206 (18704-1-AP, Proteintech), HO-1/HMOX1 (66743-1-IG, Proteintech), SPC (bs-10067R, Bioss), Elastin (ab21610, Abcam), alpha smooth muscle Actin (α-Abcam ab21027), CD34 (ab81289, Abcam), and CD31 (LS-C348736, LifeSpan BioSciences). Microscopic fluorescence images were digitized using an LSM 710 (Carl Zeiss, Oberkochen, Germany) confocal microscope.

**Statistical analysis**

All values are expressed as means ± SD for 3 independent experiments. The data were analyzed by the Tukey-Kramer method when analysis of variance (ANOVA) indicated a significant difference between concentrations (**P* < 0.05 or ***P* < 0.01).

**Table E1. RT- PCR primers**

| Gene | Sense primer 5′–3′ | Antisense primer 5′–3′ |
| --- | --- | --- |
| hGAPDH | GGAGTCCACTGGCGTCTTCAC | GCTGATGATCTTGAGGCTGTTGTC |
| hVEGF | GGCCTTCGCTTACTCTCACC | CTGTCATGGGCTGCTTCTTC |
| hVEGFR-1 | CAGGCCCAGTTTCTGCCATT | TTCCAGCTCAGCGTGGTCGTA |
| hVEGFR-2 | CCAGCAAAAGCAGGGAGTCTGT | TGTCTGTGTCATCGGAGTGATATCC |
| hPDGFα | GCAAGACCAGGACGGTCATTT | GGCACTTGACACTGCTCGT |
| mGAPDH | TTGAGGTCAATGAAGGGGTC | TCGTCCCGTAGACAAAATGG |
| miNos | CCAAGCCCTCACCTACTTCC | CTCTGAGGGCTGACACAAGG |
| mTRAP | TGGTCCAGGAGCTTAACTGC | GTCAGGAGTGGGAGCCATATG |
| mTNFα | GACGTGGAACTGGCAGAAGAG | GCCACAAGCAGGAATGAGAAG |
| mIL-6 | CCACTTCACAAGTCGGAGGCTTA | GCAAGTGCATCATCGTTGTTCATAC |
| mIL-1β | ATCTCGGAGCCTGTAGTGC | CCACTTCACAAGTCGGAGGCTTA |

mouse (m), human (h)

**Table E2.** **Patient characteristics**

| Case | Age | Gender | Date of sampling |
| --- | --- | --- | --- |
| 1 | 79 | M | 2021.6.22 |
| 2 | 80 | F | 2021.3.9 |
| 3 | 84 | M | 2021.3.11 |
